# Interplay between up-regulation of cytochrome-c-oxidase and hemoglobin oxygenation induced by near-infrared laser

**DOI:** 10.1038/srep30540

**Published:** 2016-08-03

**Authors:** Xinlong Wang, Fenghua Tian, Sagar S. Soni, F. Gonzalez-Lima, Hanli Liu

**Affiliations:** 1 Department of Bioengineering, the University of Texas at Arlington, 500 UTA Blvd, Arlington, TX 76010, USA; 2 Department of Psychology and Institute for Neuroscience, the University of Texas at Austin, 108 E. Dean Keeton Stop A8000, Austin, TX 78712, USA.

## Abstract

Photobiomodulation, also known as low-level laser/light therapy (LLLT), refers to the use of red-to-near-infrared light to stimulate cellular functions for physiological or clinical benefits. The mechanism of LLLT is assumed to rely on photon absorption by cytochrome c oxidase (CCO), the terminal enzyme in the mitochondrial respiratory chain that catalyzes the reduction of oxygen for energy metabolism. In this study, we used broadband near-infrared spectroscopy (NIRS) to measure the LLLT-induced changes in CCO and hemoglobin concentrations in human forearms *in vivo*. Eleven healthy participants were administered with 1064-nm laser and placebo treatments on their right forearms. The spectroscopic data were analyzed and fitted with wavelength-dependent, modified Beer-Lambert Law. We found that LLLT induced significant increases of CCO concentration (Δ[CCO]) and oxygenated hemoglobin concentration (Δ[HbO]) on the treated site as the laser energy dose accumulated over time. A strong linear interplay between Δ[CCO] and Δ[HbO] was observed for the first time during LLLT, indicating a hemodynamic response of oxygen supply and blood volume closely coupled to the up-regulation of CCO induced by photobiomodulation. These results demonstrate the tremendous potential of broadband NIRS as a non-invasive, *in vivo* means to study mechanisms of photobiomodulation and perform treatment evaluations of LLLT.

Low-level laser/light therapy (LLLT), also known as photobiomodulation, refers to the use of low-level light in the red-to-near-infrared range (620–1100 nm) to stimulate cellular functions for physiological or clinical benefits. Photobiomodulation has been used to improve wound healing^1^,^2^, reduce pain^3^,^4^, and many other human applications. The light can be supplied by lasers or light-emitting diodes (LEDs). In recent years, transcranial LLLT has gained increased recognition for its therapeutic use in various neurological and psychological conditions, including ischemic stroke^5^,^6^, chronic traumatic brain injuries^7^,^8^, and depression^9^,^10^. Furthermore, using a 1064-nm laser, Barrett and Gonzalez-Lima conducted the first placebo-controlled studies demonstrating that LLLT to the forehead benefits cognition in healthy humans, including enhanced attention, working memory, and executive functions^11^,^12^,^13^.

The mechanism of photobiomodulation is proposed to rest on photon absorption by cytochrome c oxidase (CCO)^14^, the terminal enzyme in the mitochondrial respiratory chain that catalyzes the reduction of oxygen for energy metabolism^1^,^2^,^15^,^16^. The more the activity of CCO increases, the more oxygen consumption and metabolic energy is produced via mitochondrial oxidative phosphorylation^17^. Since CCO is an inducible enzyme, a longer-lasting metabolic effect is achieved by LLLT’s up-regulating CCO concentration, which in turn enhances the capacity for cellular oxygen metabolism [13]. Because neurons are cells highly dependent on oxygen metabolism, this photonics-bioenergetics mechanism results in metabolic and hemodynamic alterations that facilitate neuronal functioning^15^,^18^. To date, most research into effects of LLLT on mitochondrial enzymes has been conducted in cultured neurons^1^,^2^ and animal brains^19^ with invasive means. There is lack of experimental or direct observation on how LLLT modulates CCO levels and how the up-regulated enzyme affects or interplays with hemodynamic oxygenation in human tissues *in vivo*. The primary goal of this study was to utilize an experimental optical imaging approach to observe LLLT-induced up-regulation of CCO and its relationship with hemoglobin oxygenation in human forearms for better understanding and validation of photobiomodulation *in vivo* effects.

Near-infrared spectroscopy (NIRS)^20^ is a non-invasive and portable technology that can be used to probe biological and physiological states of living tissues based on the level of absorption and scattering of near-infrared light. In the past two decades, NIRS has been broadly investigated for quantification of oxygenated and deoxygenated hemoglobin concentrations (i.e., [HbO] and [Hb], respectively) in a variety of tissues^21^, such as the human breast^22^,^23^,^24^, the human prostate^25^,^26^, and the human brain^24^,^27^, in order to diagnose cancers or to map/image functional brain activities *in vivo*. In conventional NIRS, two or three wavelengths are adequately employed for characterizing cerebral or tissue HbO and Hb concentrations under different stimulations or psychological conditions. Based on the same working principle, we have recently utilized dual-wavelength NIRS to assess the hemodynamic effects of transcranial LLLT in the human brain *in vivo*. We found that transcranial 1064-nm laser improved cerebral oxygenation, indicated by an increase of [HbO] and a decrease of [Hb] in a dose-dependent manner^28^. However, because of the limited number of measuring wavelengths in dual-wavelength NIRS, we were not able to reliably quantify any change or elevation of CCO. As the mechanistic action of LLLT relies on direct photoactivation of CCO, it is crucial to quantify the LLLT-induced CCO changes as well. Thus, further improvement in our methodology was sought in order to address this critical need.

Since the initial development of NIRS technology, a significant amount of research effort has been persistently made to utilize broadband NIRS (bb-NIRS) for calculating the redox state of CCO based on its absorption band at 820–840 nm^29^. While the actual implementation of this approach started more than 20 years ago^30^,^31^, it had uncertainty on the accuracy of the methodology^29^,^32^,^33^. It is only in recent years when bb-NIRS has been reported by numerous publications to be a reliable means for computing both cerebral hemoglobin and CCO concentration changes during brain activations^34^,^35^,^36^ and/or brain injury^37^,^38^. Therefore, bb-NIRS has become a unique and valid tool to facilitate our measurement of LLLT-induced up-regulation of CCO and its relationship to the alteration of hemoglobin oxygenation in treated tissues. We investigated human forearms as a model to reduce tissue heterogeneity during photobiomodulation and to avoid the complication of extra-cerebral layers (i.e., the human scalp and skull). The current study applied LLLT on human forearms using a 1064-nm laser and interleaved the bb-NIRS data acquisition *in vivo* for simultaneous assessment of interplay between photoactivation/up-regulation of CCO and alteration of hemoglobin oxygenation of the treated tissue.

## Results

**Figure 1:**
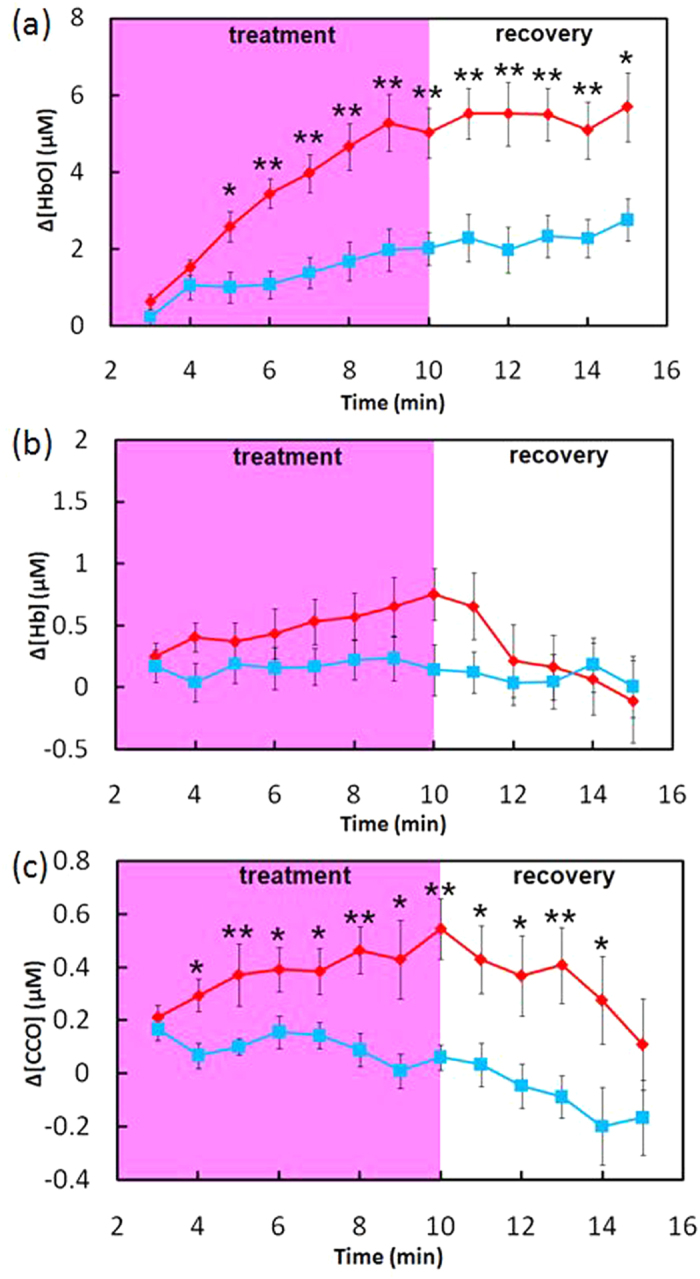
LLLT (red)/placebo (blue)-induced concentration changes of (**a**) [HbO], (**b**) [Hb], and (**c**) [CCO] in human forearms *in vivo* (mean ± SE, n = 11). In each subplot, the pink-shaded region indicates the period of LLLT/placebo treatment; *indicates significant differences in respective concentrations between LLLT and placebo treatment (0.01 < p < 0.05, paired t-test). **indicates significant differences in respective concentrations between LLLT and placebo treatment (p < 0.01, paired t-test).

A total of 11 normal subjects (seven males and four females, mean ± SD age = 26.1 ± 5.0 years) participated in the experiments. Figure 1 shows dose-dependent (energy density dose = exposure time × laser power density) concentration changes in [HbO], [Hb], and [CCO] induced by the LLLT and placebo treatments at the group level (mean ± SE, n = 11). Overall, LLLT significantly increased the HbO and CCO concentrations as compared with placebo (0.01 < p < 0.05 and p < 0.01), while the Hb concentration was nearly unaltered by either placebo or laser treatment. However, the initial laser effect on CCO seemed to precede in time the effect on HbO; i.e., for [HbO] the laser-induced effect was significantly greater than placebo after two minutes of laser treatment [Fig. 1(a)], whereas for [CCO] the significant effect started after one minute [Fig. 1(c)]. Also note that the increased CCO concentration showed a slightly faster recovery trend towards the baseline than HbO after LLLT.

**Figure 2:**
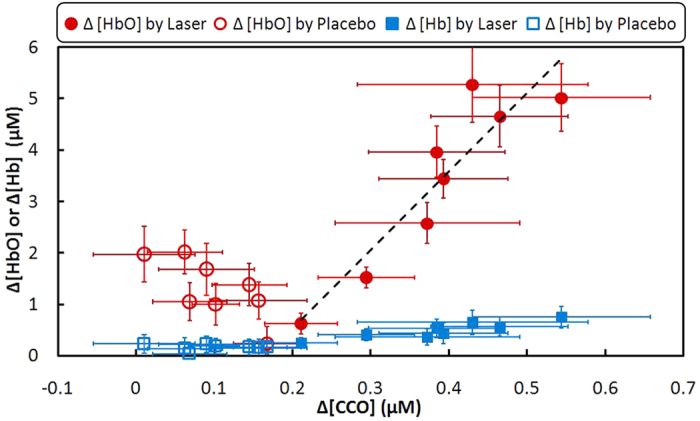
The relationship of concentration changes between CCO vs. HbO or Hb during LLLT and placebo experiment (mean ± SE, N = 11). The horizontal error bars represent variability of CCO and the vertical error bars represent variability of HbO or Hb. The correlation coefficient of the fitted line is *r* = 0.92 with a *p* value of 0.001.

After close inspection of the data in Fig. 1, we reorganized the respective concentrations and replotted them to show the relationship between concentration increases of CCO vs. HbO or Hb, induced by either LLLT or placebo treatment. As shown in Fig. 2, the solid red dots display the relationship of Δ[CCO] vs Δ[HbO] during LLLT, with a linear fit by the dashed line (with a correlation coefficient of *r *= 0.92 and a *p* value of 0.001), confirming an excellent linear relationship between them. On the other hand, the open red circles, obtained under the placebo treatment, were gathered within a lower Δ[CCO] range with no relationship between Δ[CCO] and Δ[HbO]. The concentration change of Hb also showed no response to the change of CCO, as plotted by the blue squares in Fig 2.

## Discussion

In this placebo-controlled study, we used broadband NIRS to measure the LLLT-induced changes or increases in oxygenated hemoglobin and CCO concentrations in human forearms *in vivo*. For the first time, we demonstrated that 1064 nm laser can induce significant increases of CCO and HbO concentrations in a dose-dependent manner over time, as compared with placebo treatment. In addition, Δ[CCO] and Δ[HbO] displayed a clear linear relationship as the dose of LLLT increased. Especially, we carefully measured and quantified the wavelength-dependent DPF factor, *DPF*(*λ*) as given by Eq. (5), to minimize crosstalk artifacts^35^,^36^. To the best of our knowledge, this is the first study to assess the CCO enzyme up-regulation effects of photobiomodulation in human tissues *in vivo*. These results demonstrate the great potential of bb-NIRS as a non-invasive technology for mechanistic studies and treatment evaluations of LLLT.

### Interplay between up-regulation of CCO and hemoglobin oxygenation induced by LLLT

The observed linearity between Δ[CCO] and Δ[HbO] induced by LLLT is of great significance, showing a close interplay between the up-regulation of CCO and corresponding hemodynamic oxygenation in the treated tissue. As compared to placebo, the infrared laser treatment induced a significant increase in [CCO] that preceded the increase in [HbO]. Together these data suggest that laser-induced CCO up-regulation leads to a linear increase in HbO. This may indicate that a hemodynamic oxygenation response occurs *in vivo* as a result of up-regulation of CCO induced by the infrared laser treatment. The mechanism of the observed effects can be explained based on what is known about the role of CCO on photobiomodulation^2^,^39^ and the three main steps in cell respiration: glycolysis, Krebs cycle and the electron transport chain. During the first two steps, comparatively little amount of ATP is synthesized. High energy electrons are stored in NADH/FADH; CO_2_ and water are produced as waste products. In the electron transport chain, where the most amount of energy is produced, CCO (as the terminal enzyme) transfers electrons to enable an oxygen molecule to combine with protons and form a water molecule. At the same time, this process accompanies ATP synthesis (oxidative phosphorylation). Since CCO is the main photo-acceptor within the effective optical window of LLLT, up-regulation of CCO will boost the electron transport, up-regulate the enzymatic activity, and result in a significant increase in oxygen consumption rate within tissue mitochondria. Consequently, an increase in hemodynamic oxygen supply and total blood volume will occur around the LLLT area due to the need for more oxygen and electrons. Therefore, during LLLT, the more the redox state of CCO is activated, the more the oxygenated hemoglobin concentration, HbO, increases proportionally.

In a previous study on the hemodynamic effects of transcranial LLLT on the human brain using dual-wavelength NIRS^28^, we have reported that transcranial 1064 nm laser improved cerebral oxygenation as indicated by an increase of [HbO] and a decrease of [Hb]. In the current study, we have consistently observed LLLT-induced increases of [HbO] in the human forearms, but [Hb] remained nearly unchanged. A couple of factors may be attributed to this discrepancy in the alteration of [Hb] between the two studies. The first factor can result from the large difference in anatomy and physiology between the human forearm and the brain. The brain is much more vascularized than the forearm, and it has a much greater rate of tissue oxygenation. This may lead to a greater differential hemoglobin concentration in the LLLT-stimulated brain region, with a relative [HbO] increase and [Hb] decrease [24]. The second factor may stem from differences in experimental setups and quantification algorithms that were used to measure and quantify changes in [HbO] and [Hb]. In our previous study, a dual-wavelength NIRS system was used. In consequence, changes in [HbO] and [Hb] were determined using the dual-wavelength-based, modified Beer-Lambert law with a fixed pathlength factor (i.e., DPF is independent of wavelength)^28^. While the use of a constant DPF is a common practice in the NIRS field, the derived quantifications of Δ[HbO] and Δ[Hb] are more likely subject to cross-talk errors due to the inaccurate assumption of constant pathlength^32^,^33^, particularly when changes of [CCO] are involved. In the current study, on the other hand, we employed a bb-NIRS system, carefully determined wavelength-dependent DPF values, and fitted the measured optical density spectra with a more rigorous expression of Eq. (4) using linear regression analysis. All of these procedures, in principle, should minimize cross-talk errors and lead to improved accuracy of Δ[HbO] and Δ[Hb] determination^35^,^37^. A limitation of this study for transcranial LLLT applications is that the LLLT-induced linear interplay between Δ[CCO] and Δ[HbO] was demonstrated on the human forearm, but not on the brain. Our future work plans to follow similar LLLT experimental protocols and perform bb-NIRS measurements on the human forehead in order to confirm in the brain the findings reported in this paper.

### Rationale of using 1064-nm laser for photobiomodulation

It is noteworthy that the CCO enzyme effects of LLLT are dependent on the wavelength of the stimulation laser (light). A previous study on cultured neurons^2^ has shown that the most effective wavelengths paralleled the near-infrared absorption peaks of CCO. The current study used a 1064-nm laser that was also employed in our previous studies^11^,^12^,^28^. This wavelength may not be optimal for photon absorption by CCO because its known peaks of light absorption are at lower wavelengths^39^. However, none of the previous absorption studies have measured photon absorption by CCO at 1064 nm, and the present study demonstrated a clear effect of this wavelength on CCO up-regulation. The primary reason for selecting this wavelength is its ability to better penetrate the human scalp and skull in transcranial applications. In biological tissues, light scattering is the dominant light-tissue interaction, and its influence is two orders of magnitude greater than that of light absorption. According to Mie theory, the light scattering in biological tissues decreases with longer wavelengths^40^. The 1064-nm wavelength used in our studies is approximately the longest one in the near-infrared optical window where water absorption remains low^40^. Therefore, 1064 nm is expected to have better penetration depth and stimulation efficiency in the human brain than shorter wavelengths in transcranial laser treatments. For more details on justification of using a 1064-nm laser, please refer to Supplementary Information Section 1.

### Measurement accuracy of bb-NIRS on CCO quantification

Quantification of the redox state of cytochrome oxidase in living tissue has been a scientific topic and continuously studied over the last 20+ years^30^,^31^,^33^,^35^,^37^,^38^,^41^. In particular, continuous development on measurement techniques and improved algorithms have been done by the research group from the University College London, which has continuously made significant efforts to validate and improve the sensitivity, specificity, and accuracy of quantified CCO sensed in living tissues. The listed references of 33,35,37,38,41 demonstrate and support the scientific basis and rigor for CCO quantification by bb-NIRS that we utilized in this manuscript.

A possible question is whether, besides CCO, there is any other target or biomarker at the cellular level that contributes to photobiomodulation by absorbing 1064 nm laser. With the current scientific knowledge available, our answer is “No”, which is based on many scientific observations reported in the last 20 or more years. A recent review paper summarized that the major biological tissues that absorb light in 700–1200 nm are blood, water, melanin, adipose tissue/fat, and yellow pigments^40^. On the other hand, we have not found much reporting in the literature that other LLLT biomarkers at the cellular level (besides CCO) absorb light at 1064 nm *in vivo*. Future scientific discovery may alter our current view, but the conclusion given in this paper holds its scientific foundation and rigor.

### Possible thermal effects of LLLT on CCO quantifications

It is reasonable to expect that infrared light at 1064 nm with a power of 3.4 W would generate some thermal effect that may lead to an increase in skin blood flow (SBF). Such an increase of SBF may give rise to an increase of hemoglobin concentration in the adjacent area surrounding or near the LLLT stimulation spot. To address this concern, we conducted a pilot study, including 4 out of the 11 subjects who participated in the LLLT/Placebo study. Please refer to Supplementary Information Section 2 and Figures for details regarding the results of this pilot study. The clear observation was that thermally induced Δ[HbO] followed a similar trend to that of the placebo trace, while the LLLT-induced Δ[HbO] remained significantly higher during and after the 8 continuous laser treatments. On the other hand, in the case of Δ[CCO], the thermal effect was non-significant on changes in CCO, while significant increases of CCO were clearly observed due to LLLT stimulations.

The overall conclusion from the pilot thermal test was that thermal effects on skin surface may be non-significant to cause changes in tissue redox CCO concentrations that are measured by bb-NIRS with a separation larger than 1.5 cm. However, our sample size for the pilot study was only 4, so this conclusion was not statistically solid, and further studies with more participants are highly desirable to confirm this finding.

In final conclusion, this study has clearly demonstrated that LLLT can induce significant increases of [CCO] and [HbO] on the human forearm as the laser energy dose is accumulated over time, as compared with the placebo treatments. A strong linear interplay between Δ[CCO] and Δ[HbO] was observed for the first time during the laser treatment, indicating a hemodynamic response of oxygen supply coupled to the increase of cellular metabolic rate induced by photobiomodulation. These results demonstrate the tremendous potential of bb-NIRS as a non-invasive optical means to study *in vivo* mechanisms and perform treatment evaluations of LLLT.

## Material and Methods

### Participants

Healthy human participants were recruited from the local community of The University of Texas at Arlington. Interested individuals were screened by one of the investigators to determine whether they were eligible for the study. The inclusion criteria included: either sex, any ethnic background, and in an age range of 18–40 years old. The exclusion criteria included: (1) diagnosed with a psychiatric disorder, (2) history of a neurological condition, or severe brain injury, or violent behavior, (3) have ever been institutionalized/imprisoned, (4) current intake of any medicine or drug, or (5) currently pregnant. In addition, none of the participants were smokers or had diabetes. Eligible participants underwent two separate experiments in sequence: in the first experiment, placebo treatment was administered on their right forearms. In the second experiment, LLLT was administered on the same location as in the placebo treatment, 5 min after the first experiment. The study protocol was approved by the institutional review board (IRB) at The University of Texas at Arlington and complied with all applicable federal and NIH guidelines. Informed consent was obtained from each participant prior to the experiments.

### Instruments

Both placebo and laser treatments were administered with a continuous-wave, 1064-nm laser provided by Cell Gen Therapeutics LLC, Dallas, TX (Model CG-5000). This laser is an FDA-cleared device for various uses on humans, such as relief of muscle and joint pain. It had a hand-held aperture with a button on the handle to open and shut the laser beam. The area of laser beam from the aperture was 13.6 cm^2^. Contact delivery is relevant when laser beams are divergent and not well collimated. But in our case, the laser was well collimated, so the laser beam size did not change significantly between the laser aperture and the stimulation spot on the subject’s forearm. The non-contact delivery distance was about 2 cm with possible variation of a few millimeters because of the handheld setting. However, such a distance variation did not result in dose fluctuation in laser radiation due to excellent laser collimation. For the laser treatment, the device was operated at a constant power of 3.4 W. The irradiance (or power density) in the beam area was 0.25 W/cm^2^, the same as that used in our previous studies^11^,^12^,^28^. For the placebo treatment, the same device was operated at a minimal power of 0.1 W and the aperture was further covered up by black tapes so that no light came out from the covering tapes. Thus the actual laser power of placebo was zero.

While we used a FDA-cleared Class 4 infrared laser (International standard IEC 60825-1), this laser was used at a lower power density corresponding to that of a Class 3b laser to avoid potential skin damage. The power density used was 0.25 W/cm^2^ (whereas over 0.5 W/cm^2^ is used for Class 4 classification). Following previously successful studies with full IRB approvals, our safe laser stimulation parameters were calculated as follows:

Total laser power = 3.4 W;

Area of laser beam radiation = 13.6 cm^2^;

Power density = 3.4 W/13.6 cm^2^ = 0.25 W/cm^2^;

Time radiated per cycle = 55 s;

Total laser energy dose per cycle = 3.4 W × 55 s = 187 J/cycle.

If another laser with a smaller beam size is used, the total laser power should be adjusted in order to maintain the same safe low power density of 0.25 W/cm^2^ and thus avoid potential skin damage.

**Figure 3:**
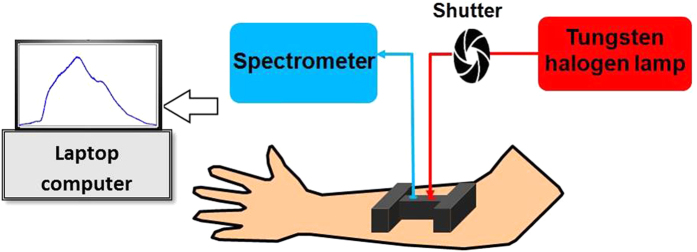
Schematic diagram of the experimental setup, including the broadband NIRS system. This bb-NIRS consisted of a tungsten halogen lamp as light source and a miniature back-thinned CCD spectrometer as detector. A laptop computer was used to acquire, display and save the data from the spectrometer. The shutter controlled the on and off of the white light from the tungsten halogen lamp to the tissues.

A single-channel, bb-NIRS system was constructed to measure changes of hemoglobin and CCO concentrations *in vivo* in LLLT and placebo experiments. As shown in Fig. 3, this system consisted of a tungsten halogen lamp (Model 3900, Illumination Technologies Inc., East Syracuse, NY) as light source and a miniature back-thinned CCD spectrometer (*i*-trometer, B&W Tek Inc., Newark, DE) as light detector, in the spectral range of 450–1100 nm. Broadband white light from the lamp was relayed by an optical fiber bundle of 3.5-mm in diameter to a shutter and then to an I-shaped optical probe holder that was placed on each subject’s right forearm. The diffuse light through the arm tissue was collected by another fiber bundle held by the same probe holder and then relayed to the spectrometer. The distance between the source and detector fiber bundles was 1.5 cm. A laptop computer was used to acquire, display and save the data from the spectrometer. The shutter controlled the on and off of the white light delivered to the tissues.

**Figure 4:**
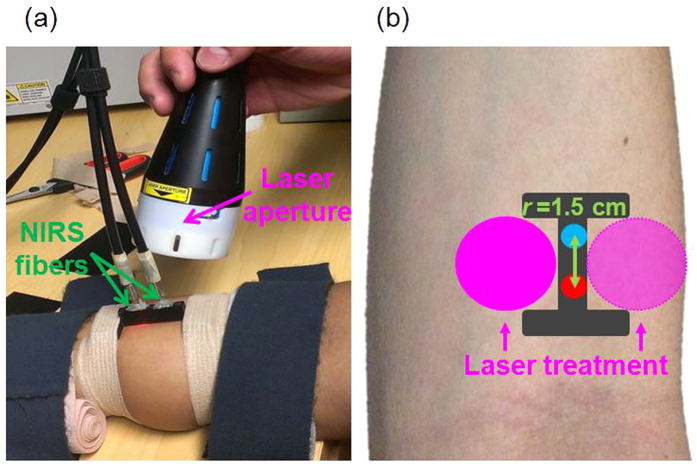
Experimental setup: (**a**) photograph of the laser aperture for LLLT/placebo treatment and bb-NIRS fiber holder on a participant’s forearm. (**b**) Configuration of the I-shaped bb-NIRS probe holder (dark gray). The bundle holder held two optical fiber bundles with a separation of 1.5 cm. One bundle (in red) was connected to the tungsten halogen lamp and the other (in blue color) to the spectrometer. The LLLT/placebo treatments were administered on two sides of the middle section alternatively (pink circles).

In particular, the I-shaped fiber bundle holder was designed using SolidWorks (SolidWorks Corp., USA) and 3D printed with solid, black material. The two wider ends of the holder were firmly fastened on each participant’s right forearm with elastic bandages (see Fig. 4a). As each participant might have slight body movements during the corresponding experiment, this experimental setup minimized potential motion artifacts during data acquisition. The narrow, middle section of the holder is ~8 mm in width. In both experiments, the laser beam from CG-5000 was administered on both sides of this section alternatively (see Fig. 4b).

### Experiments

**Figure 5:**
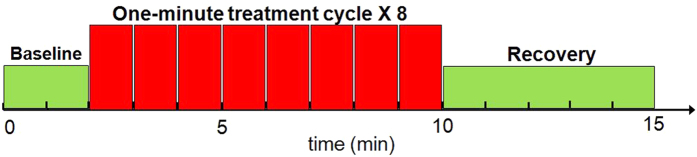
Paradigm of the LLLT/placebo treatment and interleaved bb-NIRS data acquisition. Each treatment session consisted of eight one-minute treatment cycles, 55-s laser on and 5-s laser off per cycle. The bb-NIRS data acquisition was initiated two minutes before the first treatment session and ceased five minutes after the treatment session.

The experiments were conducted in a locked room without any reflective surface. The background light from outside of the room was minimized by covering the windows and door slits with black curtains. Furthermore, when the laser was in use, a warning sign of “Laser on” was shown on the outer door. Protective goggles (900–1000 nm: 5+, 1000–2400 nm: 7+; 2900–10600 nm: 7+) were worn by all individuals present in the room. The participants were further instructed to close eyes during the treatments. After each participant was comfortably seated, an experimenter first measured the absorption coefficient (*μ*_*a*_) and reduced scattering coefficient (*μ*_*s*_′) at 750 nm and 830 nm from the participant’s right forearm using a frequency-domain NIRS tissue oximeter (OxiplexTS, ISS Inc., Champaign, IL). Then the I-shaped optical probe holder was placed on the same location. A trained experimenter held the aperture of CG-5000 laser closely to the participant’s right forearm to administer the placebo or LLLT treatment on two sides of the holder alternatively. As illustrated in Fig. 5, each treatment session consisted of eight one-minute cycles, 55-s laser on and 5-s laser off per cycle. The participant was given a 2-minute break between the two experiments.

The participants received no information about the treatment type (placebo or LLLT) in each experiment. Instead, they were instructed that they would receive the same laser treatment at a power of 3.4 W in both experiments. Furthermore, the laser at a power of 3.4 W generated negligible heat on the participants’ skin. Thus, the two experiments were designed to cause approximately the same sensations and expectations in the participants.

The data acquisition of bb-NIRS was initiated two minutes before each treatment session and ceased five minutes after the treatment session. Because the power of treatment laser from CG-5000 was high enough to contaminate the bb-NIRS readings, data acquisition interleaved with the treatment cycles during the 5-s laser-off periods. Following the similar format/fashion, the data during pre-treatment baseline and post-treatment recovery were also acquired at 55-second interval. In this way, a total of 15 data points (see Fig. 5) were obtained throughout each experiment. The shutter was switched on only for 5 seconds during each data acquisition period and then off in the rest of the time.

### Theoretical Foundation for Data Processing and Error Analysis

Raw data from the broadband spectrometer was processed using MATLAB to calculate relative changes in [HbO], [Hb] and [CCO] from the initial baseline. First, the relative optical density, ΔOD, was calculated at each wavelength, λ:
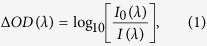
where *I*_0_(*λ*) is the spectral data acquired at the initial baseline (i.e., the first spectrum collected in each experiment), and *I*(*λ*) is the data acquired at each time point thereafter.

According to the Modified Beer-Lambert Law^21^,^42^, Δ*OD* at each *λ* could be expressed as a sum of optical absorbance contributed by HbO, Hb and CCO components:
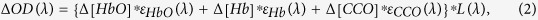
where Δ[HbO] is the relative change in HbO concentration, Δ[Hb] is the relative change in Hb concentration, Δ[CCO] is the relative change in CCO concentration, ε_HbO_ (*λ*), ε_Hb_(*λ*) and ε_CCO_(*λ*) are the extinction coefficients of HbO, Hb and CCO, which can be found in ref. 35, and *L*(*λ*) denotes the effective pathlength of the detected photons through the tissues. According to the Modified Beer-Lambert Law^21^,^42^, *L*(*λ*) can be estimated as:
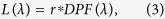
where *r* is the source-detector distance, and *DPF*(*λ*) is the wavelength-dependent differential pathlength factor. Note that in this study, we did not assume that *DPF* was a wavelength-independent constant across the wavelength range. By substituting Eq. (3) into Eq. (2) for multiple wavelengths, we can express Δ[HbO], Δ[Hb] and Δ[CCO] in a matrix format in association with broadband *ΔOD*(*λ*) over *DPF*(*λ*), as follows:
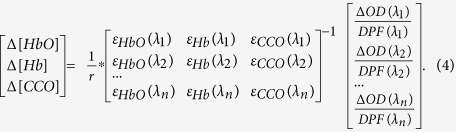


In this study, the *DPF*(*λ*) values were estimated based on the *μ*_*a*_ and *μ*_s_′ values measured with a frequency-domain OxiplexTS tissue oximeter in the beginning of the experiments. In principle, an OxiplexTS tissue oximeter provides measurement readings of *μ*_a_ and *μ*_s_′ values at 750 nm and 830 nm as well as absolute concentrations of [HbO] and [Hb]. To achieve *μ*_*a*_ and *μ*_s_′ values across the entire wavelength range of 740–900 nm, we interpolated and extrapolated the two measured *μ*_s_′ values at 750 nm and 830 nm by following Mie theory, which is usually expressed by *kλ*^*−b*^, where *k* and *b* were obtained by fitting this equation to both *μ*_s_′(750 nm) and *μ*_s_′(830 nm). In the meantime, the absorption coefficients in the same wavelength range (740–900 nm) were estimated based on the HbO and Hb concentrations measured by the same tissue oximeter. Then, the wavelength-dependent *DPF* values were calculated using the diffusion theory with the semi-infinite boundary geometry^43^:
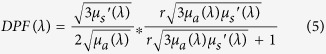
where *μ*_a_(*λ*) and *μ*_s_′(*λ*) are the estimated absorption and reduced scattering coefficients across the wavelength range of interest.

Next, the final and key step was to quantify or determine three chromophore concentrations based on Eq. (4). To do so, multiple linear regression analysis was implemented in the wavelength range of 740–900 nm (with a total of 161 wavelengths) using a MATLAB-based function. This regression algorithm afforded the best fit of the chromophore-specific concentrations to the measured *ΔOD*(*λ*) spectrum by minimizing the squared residual or the objective function. The detailed fitting procedures are given next.

### Multiple Linear Regression Analysis

**Figure 6:**
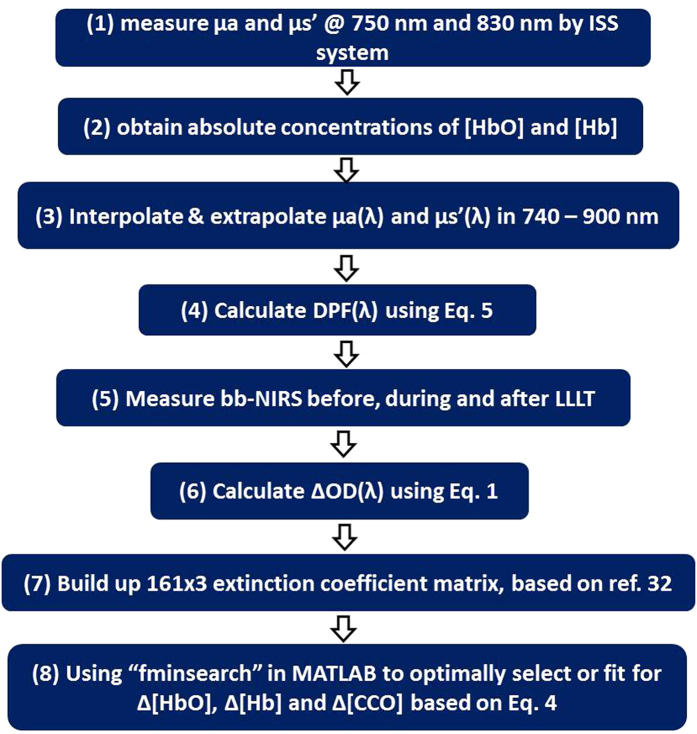
A flow chart describing detailed procedures of our multiple linear regression analysis to optimally determine LLLT-induced concentration changes in three chromophores.

The procedure for multiple linear regression analysis is outlined with a flow chart in Fig. 6.(1)

(1) Start data collection and quantification of *μ*_*a*_ and *μ*_s_′ values at 750 nm and 830 nm of the subject’s right forearm by the OxiplexTS tissue oximeter.(2)

(2) Obtain output readings from the oximeter for absolute concentrations of [HbO] and [Hb].(3)

(3) Interpolate and extrapolate *μ*_*a*_(*λ*) and *μ*_*s*_′(*λ*) values across 740 nm to 900 nm, based on *μ*_*a*_ and *μ*_s_′ values at 750 nm and 830 nm, by fitting Mie theory (*kλ*^*−b*^) and calculating [HbO] and [Hb] with their corresponding extinction coefficients given in ref. 35.(4)

(4) Calculate *DPF*(*λ*) values across 740 nm to 900 nm for each wavelength with Eq. (5);(5)

(5) Perform bb-NIRS experiments to acquire optical spectra (with 161 wavelengths from 740–900 nm) from the subject’s arm before, during and after LLLT (or placebo);(6)

(6) Quantifying *ΔOD*(*λ*) at different time points, using Eq. (1), to form a time series for each wavelength.(7)

(7) Build up or form the 161×3 extinction coefficient matrix, based on ref. 35; this matrix would list extinction coefficients at 161 wavelengths (740–900 nm) for 3 chromophores.(8)

(8) Solve accurately Δ[HbO], Δ[Hb] and Δ[CCO] based on Eq. (4). For the last step, specifically, we applied the MATLAB function of “fminsearch” to find/fit for the optimal combination of Δ[HbO], Δ[Hb] and Δ[CCO] as the final output parameters. In theory, “fminsearch” function finds the minimum of a scalar function (often called the objective function) of several variables, starting at an initial estimate (http://www.mathworks.com/help/optim/ug/fminsearch.html). This is generally referred to as unconstrained nonlinear optimization. In our study, for example, “fminsearch” attempted to model the relationship between three explanatory variables (e.g., Δ[HbO], Δ[Hb] and Δ[CCO]) and a set of response variables (e.g., *ΔOD* at multi-wavelengths) by fitting a linear equation to observed data, as expressed in Eq. (4). This function tries different combination of Δ[HbO], Δ[Hb] and Δ[CCO] starting from the “initial guess” to match with the measured set of *ΔOD*(*λ*) across the spectra until a minimized value of the objective equation is achieved. Then, this set of values (Δ[HbO], Δ[Hb] and Δ[CCO]) are considered as the “best fit” and to be used as the final results.

### Statistical Analysis

To determine whether the LLLT induced significant changes in hemoglobin and CCO concentrations with respect to the placebo treatment, paired *t*-test between these two treatment types was conducted for each chromophore (HbO, Hb and CCO) at each time point. A two-tailed level of 0.01 < *p* < 0.05 and p < 0.01 was chosen to be statistically significant in these tests.

## Additional Information

**How to cite this article**: Wang, X. *et al*. Interplay between up-regulation of cytochrome-c-oxidase and hemoglobin oxygenation induced by near-infrared laser. *Sci. Rep.* **6**, 30540; doi: 10.1038/srep30540 (2016).

## Supplementary Material

Supplementary Information
