# Supplementary material for: Interplay between up-regulation of cytochrome-c-oxidase and hemoglobin oxygenation induced by near-infrared laser
Source: Sci Rep. 2016 Aug 3;6:30540. doi: 10.1038/srep30540 (PMC4971496; doi:10.1038/srep30540)
Supplement: Supplementary Information [file srep30540-s1.pdf]

Supplementary Information for

Interplay between up-regulation of cytochrome-c-oxidase and hemoglobin oxygenation induced by near-infrared laser

Xinlong Wang[1#], Fenghua Tian[1#], Sagar S. Soni[1], F. Gonzalez-Lima[2*], Hanli Liu[1*]

[1]Department of Bioengineering, the University of Texas at Arlington,
500 UTA Blvd, Arlington, TX 76010
[2]Department of Psychology and Institute for Neuroscience, the University of Texas at Austin,
108 E. Dean Keeton Stop A8000, Austin, TX 78712

[#]: equal contribution

*gonzalezlima@utexas.edu; ***hanli@uta.edu**

1. Rationale to use 1064-nm laser in this study and future improvement

There are a few reasons for utilizing a 1064 nm laser. First, the absorption of water at 1064 nm is relatively weak, lower than that at 950-1000 nm and comparable to that at 900 nm (see the following figure). Second, light at this wavelength can penetrate deeper in tissue than other wavelengths shorter than 1000 nm used for LLLT because of lower light scattering. For example, 1064 nm light penetrates the human hand better than 660 nm or 980 nm, and comparable to 830 nm [data of Karl H. Norris, from The Science of Photobiology (KC Smith, ed., Plenum Press, 1977; p. 400)]. Since our future application of LLLT targets on transcranial laser stimulation or photobiomodulation, it is definitely beneficial if the light can penetrate deeper in tissue. Third, the wavelength 1064 nm is still at the upper absorption band of the redox state of CCO [1] meaning that a 1064 nm laser will be absorbed to stimulate the reduction-oxidation reaction of CCO, as we needed for this study. Fourth, this project wished to examine whether the 1064-nm laser was able to stimulate or generate a reasonable amount of changes in HbO and CCO concentrations, even using a non-optimal laser wavelength. For future studies, given the absorption spectra of water and redox state of CCO, it might be optimal to use a laser near the absorption peak of CCO (e.g., at ~830 nm) where water absorption is also lower than that at 1064 nm.

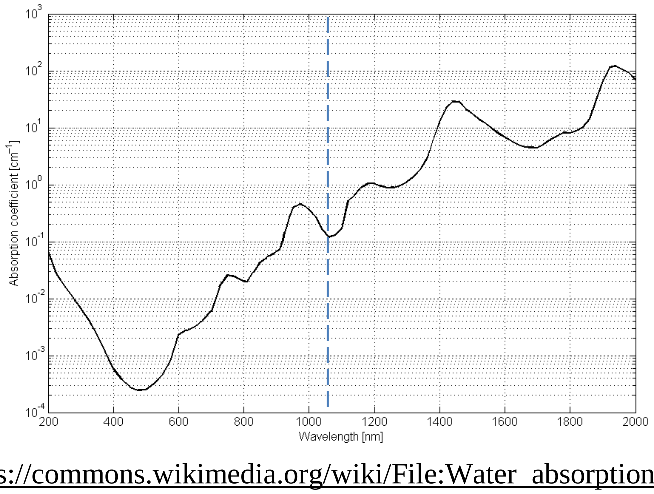

https://commons.wikimedia.org/wiki/File:Water_absorption.png

2. Possible increase of HbO and CCO induced by thermal effects

It is reasonable to expect that infrared light at 1064 nm with a power of 3.4 W would generate some thermal effect that may lead to an increase in skin blood flow (SBF). Such an increase of SBF may give rise to an increase of hemoglobin concentration in the adjacent area surrounding or near the LLLT stimulation spot. Therefore, it is essential to examine and inspect the response of HbO, Hb, and CCO concentrations to thermal stimulation.

We conducted a pilot study, including 4 out of the 11 subjects who participated in the LLLT/Placebo study. The photo/diagram shown in Supplementary Figure 1(a) illustrates a combined setup of the bb-NIRS probe and a thermal stimulator that was used to provide mild thermal stimulation. The optical measurement protocol of the bb-NIRS was kept the same as the LLLT stimulation experiment. The difference in setup between this thermal and LLLT stimulation was, instead of using laser to stimulate the arm tissue, the use of a computer-controlled thermode that was able to deliver well controlled thermal stimulation to the arm. The thermode had a contact area of 16×16 mm^2 and was connected to an ATS thermal stimulator [see Supplementary Figure 1(b)]. More information on using such a thermal stimulation can be found in ref. 2.

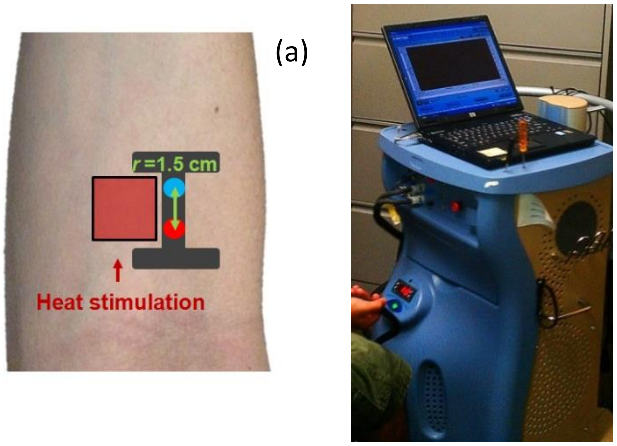

Supplementary Figure 1. (a) Photo/diagram showing the experimental setup of bb-NIRS and a thermode for thermal stimulation measurement; (b) An ATS thermal stimulator used to create accurate thermal stimulation delivered to the subject's forearm.

In this thermal stimulation test/experiment, we needed first to determine an appropriate temperature that would create a similar thermal effect to that by LLLT on the skin surface. We selected five different subjects and measured corresponding skin temperatures by a surface thermometer right after a LLLT stimulation routine. After careful comparison between several thermal settings and skin temperatures measured after LLLT, a proper temperature of 38℃ was selected for a best match to simulate thermal effects of LLLT on 5-averaged human skins.

Then, the bb-NIRS measurements were taken from 4 out of 5 subjects following the same protocol as shown in Fig. 5, except that the LLLT stimulations were replaced by the thermal stimulations at 38 ℃. The comparative results are given in Supplementary Figure 2.

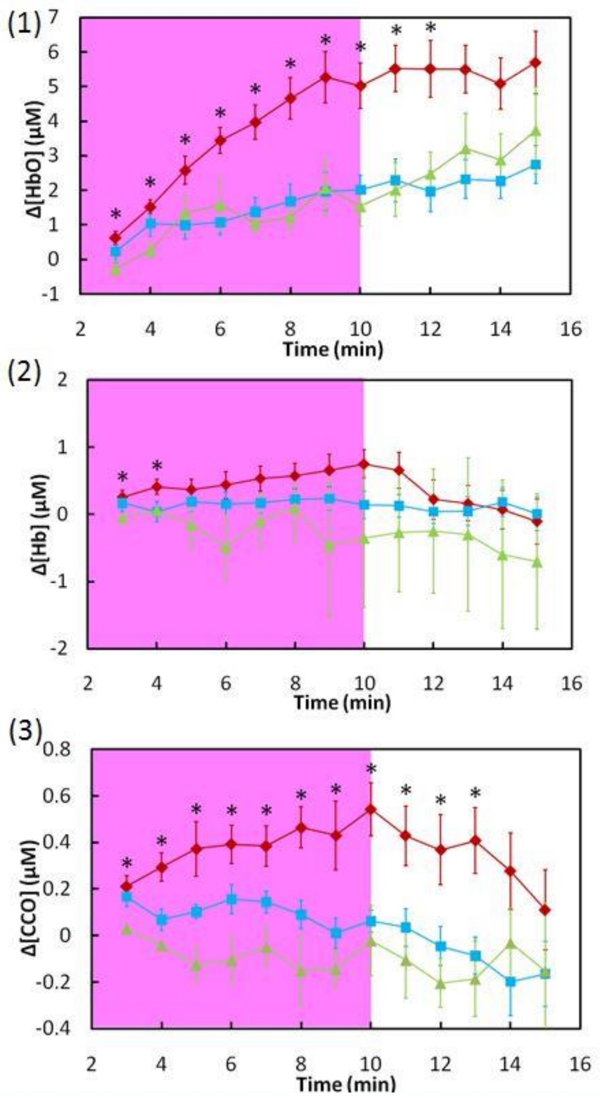

Supplementary Figure 2. Concentration changes of (1) HbO, (2) Hb and (3) CCO during LLLT (red; n=11), placebo (blue; n=11) and thermal (green; n=4) stimulation. The "*" represents significant difference between LLLT vs thermal effect (p-value <0.05).

As shown in the top panel of Supplementary Figure 2, thermally induced Δ[HbO] follows a similar trend to that of the placebo trace, while the LLLT-induced Δ[HbO] remains significantly higher during and after the 8 continuous laser treatments. On the other hand, in the case of Δ[CCO], the thermal effect is non-significant on changes in CCO, while significant increases of CCO are clearly observed due to LLLT stimulations.

Overall, the conclusion from this thermal test is that thermal effects on skin surface may be non-significant to cause changes in tissue CCO concentrations that are measured by bb-NIRS with a separation larger than 1.5 cm. However, our sample size for the thermal study was only 4, so this conclusion is not statistically solid, and further studies with more participants are highly desirable to confirm this finding.

References:

1 Kolyva, C. *et al.* Systematic investigation of changes in oxidized cerebral cytochrome c oxidase concentration during frontal lobe activation in healthy adults. *Biomedical optics express* **3**, 2550-2566, doi:10.1364/BOE.3.002550 (2012).

2 Yennu, A. *et al.* A Preliminary Investigation of Human Frontal Cortex Under Noxious Thermal Stimulation Over the Temporomandibular Joint Using Functional Near Infrared Spectroscopy. *Journal of Applied Biobehavioral Research* **18**, 22 (2013).
